# Leaf Cell Morphology Alternation in Response to Environmental Signals in *Rorippa aquatica*

**DOI:** 10.3390/ijms231810401

**Published:** 2022-09-08

**Authors:** Tomoaki Sakamoto, Shuka Ikematsu, Kazuki Namie, Hongwei Hou, Gaojie Li, Seisuke Kimura

**Affiliations:** 1 Faculty of Life Sciences, Kyoto Sangyo University, Kamigamo-Motoyama, Kita-ku, Kyoto 603-8555, Japan; 2 Center for Plant Sciences, Kyoto Sangyo University, Kamigamo-Motoyama, Kita-ku, Kyoto 603-8555, Japan; 3 The State Key Laboratory of Freshwater Ecology and Biotechnology, The Key Laboratory of Aquatic Biodiversity and Conservation of Chinese Academy of Sciences, Institute of Hydrobiology, Chinese Academy of Sciences, Wuhan 430072, China

**Keywords:** heterophylly, *Rorippa aquatica*, submergence

## Abstract

Heterophylly, the phenomenon by which plants alter leaf forms to adapt to surrounding conditions, is apparent in amphibious plant species. In response to submergence, they emerge leaves with narrower blade areas. The pathway that receives the submergence signals and the mechanism regulating leaf form via cell proliferation and/or expansion systems have not yet been fully identified yet. Our anatomical study of *Rorippa aquatica*, an amphibious plant that exhibits heterophylly in response to various signals, showed that leaf thickness increased upon submergence; this was caused by the expansion of mesophyll cell size. Additionally, these submergence effects were inhibited under blue-light conditions. The *ANGUSTIFOLIA3* (*AN3*)/*GROWTH-REGULATING FACTOR* (*GRF*) pathway regulating cell proliferation and cell expansion was downregulated in response to submergence; and the response was blocked under the blue-light conditions. These results suggest that submergence and light quality determine leaf cell morphology via the AN3/GRF pathway.

## 1. Introduction

As plants cannot avoid environmental stress by moving, forming their own tissues to adapt to the surroundings is necessary for survival. One of the responses is that leaf form alternation depends on the surrounding environmental factors, which is called heterophylly [1]. This phenomenon is often observed in amphibious plants. The water level is an important environmental factor for plants living in wetlands and changes seasonally or temporally. Narrow leaves emerge in response to submergence in various amphibious species. Narrow leaves are advantageous for deflecting the force exerted by the water flow [2,3].

*Rorippa aquatica* was used as an experimental material for studying heterophylly. This species is amphibious and emerges deeply dissected compound leaves under submerged conditions [4]. In addition, it alters leaf forms in response to various surrounding signals, such as temperature and light intensity [5]. Growth temperature remarkably affects heterophylly in this species and the underlying mechanisms were analyzed. Low temperatures induce more dissected narrow leaves under terrestrial conditions as a similar response to submergence. An anatomical study showed that low temperatures caused a decrease in leaf blade area and an increase in cell size in the sub-epidermal palisade tissue layer in mature leaves [6]. This phenomenon, in which cell size expansion arises in the background of tissue size reduction, is called “compensation” [7,8]. This may coordinate cell proliferation with cell expansion during leaf development. While submergence and low temperature have similar effects on leaf form in *R. aquatica*, anatomical changes upon submergence have not been well-studied. In the amphibious plant *Callitriche palustris* [9], epidermal and mesophyll cells became longer along the proximal–distal axis under the submerged conditions. The submerged leaves of *Hygrophila difformis* have smaller epidermal pavement cells with more lobes, resulting in a more pronounced jigsaw shape [10]. In addition, leaf cross-sections showed a clear differentiation of palisade tissue only under terrestrial conditions; and a decrease of the mesophyll layer under the submerged conditions in this species.

Light quality also affects the size and form of leaves. One of the pronounced effects of light quality on plant morphology is the shade-avoidance syndrome. The shade induces elongated petioles, leaf hyponasty and a smaller leaf blade. Low red light and far-red light-rich conditions can simulate shaded conditions. The effect of light on the submergence effect has not been studied in detail. Blue light causes the European water clover *Marsilea quadrifolia*, an amphibious fern, to emerge terrestrial leaves under submergence [11]. In *R. aquatica*, similar effects have been reported under blue-light conditions [12]. Submerged conditions with blue light lead to leaves with expanded blades, similar to terrestrial leaves. However, the effects of light on cell morphology remain unknown.

Cellular level leaf morphological changes upon submergence in *R. aquatica* have not been well-identified. We analyzed morphological changes at the cellular level and identified genetic pathways regulating leaf form alternation in response to submergence. Furthermore, the effect of the light quality on heterophylly was investigated.

## 2. Results

### 2.1. Leaf Thickness Is Affected by Submergence via Cell Size Regulation

**Figure 1:**
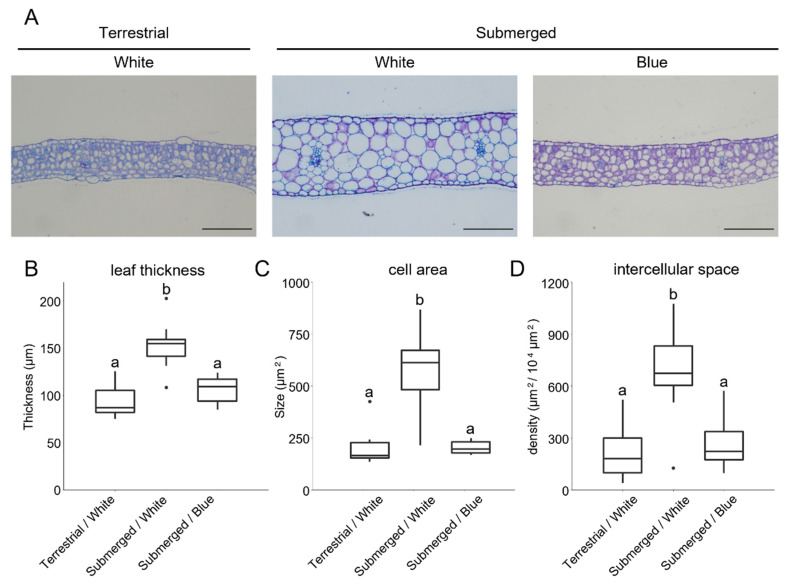
The effect of submergence upon leaf morphology and suppression under the blue-light conditions. (**A**) *R. aquatica* leaf cross-sections after 7 days of treatment (scale bars = 100 µm); a box plot of leaf thickness (**B**); the cell area size of the mesophyll cell (**C**); and the density of intercellular space (**D**) (mean ± SE, *n* = 10). The letters show the groups assigned based on the adjusted *p*-value obtained from the Tukey–Kramer test (the adjusted *p*-value < 0.01).

In order to identify the anatomical changes upon submergence, cellular characteristics were measured in young leaves of *R. aquatica*. After transfer to the submerged conditions under white light, leaf thickness was 61% greater than that of leaves grown under terrestrial conditions with white light (Figure 1). The size of the mesophyll cells was significantly larger than that of the control terrestrial conditions. The intercellular region expands under the submerged conditions. Additionally, the effects of light quality on cellular morphological changes upon submergence were analyzed. The effect on cell morphology when blue-light conditions cause the emergence of terrestrial-like leaves under submergence [12] has not yet been analyzed. Under blue light, no cellular morphological changes upon submergence were observed (Figure 1). Even under the submerged conditions, leaf thickness, cell size and intercellular area were significantly smaller in plants treated with blue light than in those treated with white light. These characteristics were similar to those observed under the controlled terrestrial conditions. These results suggest that the expansion of leaf thickness induced by submergence is regulated by at least cell-size expansion. This expansion was suppressed under blue-light conditions.

### 2.2. Cell Size Expansion upon Submergence Is Regulated through the AN3/GRF Pathway

To identify the molecular mechanism of cellular morphological changes upon submergence and suppression under blue-light conditions, the expression of genes regulating leaf form was analyzed using qPCR. The expression of *R. aquatica* homologs of *A. thaliana* genes, which were identified in *R. aquatica* genome assembly [12], were measured. The total RNA of leaves was sampled one and seven days after treatment to analyze the early responses and effects through leaf growth, respectively.

**Figure 2:**
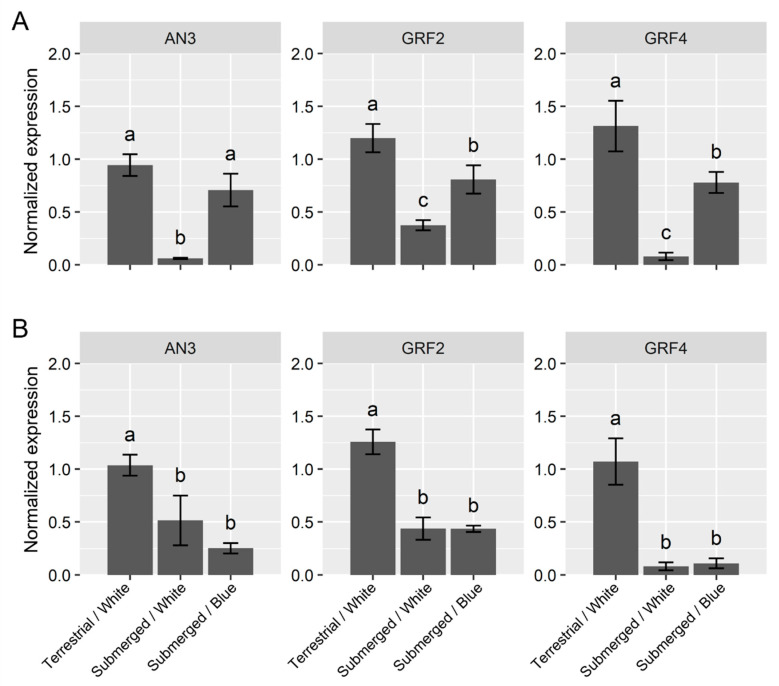
Expression profiles of cell proliferation-related genes after submergence. The plots indicate the relative expression normalized by *ubiquitin5* (mean ± SE, *n* = 3) after 1 day (**A**) and 7 days treatment (**B**). The different letters denote significant differences among the experimental conditions (Tukey–Kramer test; the adjusted *p*-value < 0.05).

First, the genes regulating cell proliferation were analyzed. The *GROWTH-REGULATING FACTOR* (*GRF*) gene family encodes a small plant-specific transcriptional factor that regulates cell purification [13]. The ANGUSTIFOLIA3 (AN3) protein interacts with GRFs and functions as a transcriptional co-activator [14]. In *Arabidopsis thaliana*, defective AN3/GRF complex activity reduced the leaf size due to a decrease in cell number. Simultaneously, the cell size was larger than that of the wild-type plants to compensate for the reduced function [15]. The AN3/GRF pathway regulates cell proliferation and expansion. After one day of submergence under white-light conditions, these cell proliferation-related genes showed significantly lower expression relative to those under terrestrial conditions (Figure 2A). After seven days, the expression levels under submergence were significantly low; however, the difference between terrestrial conditions reduced compared with that after one day of treatment in *AN3* expression (Figure 2B).

Under the blue-light conditions, one day after submergence (Figure 2A), the expression of *AN3* was slightly lower than that under the control terrestrial conditions; however, these differences were not significant. Additionally, the difference was significantly higher than that under white light upon submergence. The two *GRF* genes showed similar expression patterns. Their expression was significantly higher than that under white light submergence. However, they did not recover to the levels under terrestrial conditions. Seven days of submergence had similar effects on cell proliferation-related genes under both the white- and blue-light conditions (Figure 2B). Even under the blue-light conditions, the expression levels were significantly lower than those in terrestrial conditions.

### 2.3. Submergence Did Not Induce Polarization to Adaxial or Abaxial 

In the amphibious plant, *Ranunculus trichophyllus*, the involvement of the regulation of adaxial–abaxial polarity in leaf form alternation has been observed [16]. In this species, submergence causes upregulation of the abaxialization genes; resulting in the emergence of abaxialized narrow radial leaves. To confirm whether a similar mechanism works in the heterophylly of *R. aquatica* upon submergence, the expressions of genes coordinating adaxial–abaxial polarity were quantified.

**Figure 3:**
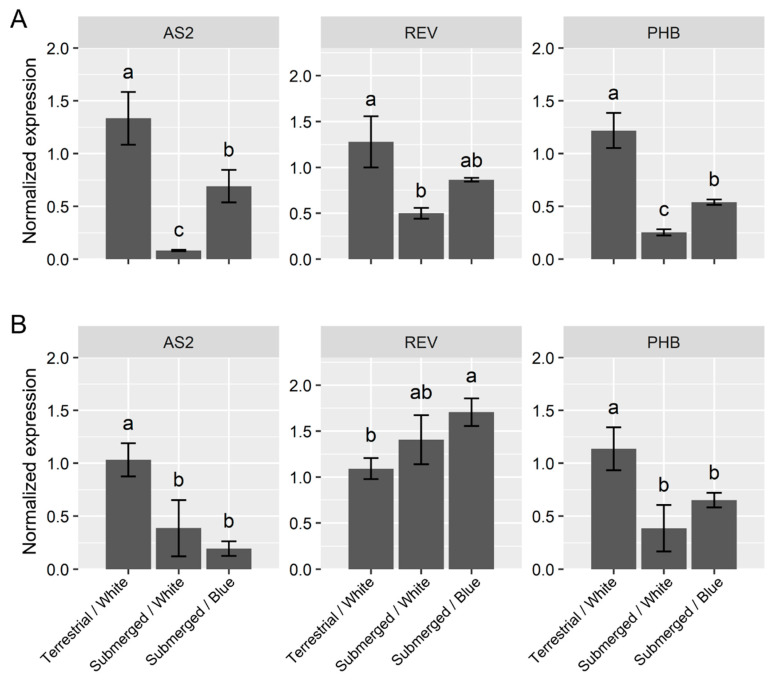
Expression profiles of the adaxialization genes after submergence. The plots indicate the relative expression normalized by *ubiquitin5* (mean ± SE, *n* = 3) after 1 day (**A**) and 7 days treatment (**B**). The different letters denote significant differences among the experimental conditions (Tukey–Kramer test; the adjusted *p*-value < 0.05).

After one day of submergence under white light, the expressions of the adaxialization genes (*ASYMMETRIC LEAVES* (*AS2*), *REVOLUTA* (*REV*) and *PHABULOSA* (*PHB*)) were lower than those under terrestrial conditions (Figure 3A). The blue-light treatment also suppressed the effect of submergence for one day for adaxialization genes. For *AS2* and *PHB*, these expression levels were significantly higher than those in the control terrestrial conditions; and the expression of *REV* tended to increase under the blue-light conditions. However, blue light could not completely suppress the effects of submergence. After seven days of submergence with white light, the adaxialization genes *AS2* and *PHB* were repressed in a manner similar to those after one day of submergence (Figure 3B). On the other hand, *REB* expression was slightly higher than that in terrestrial conditions. Blue light had no significant effect on these genes. Seven days of submerged blue-light conditions showed no differences from the adaxialization genes under the white-light submerged conditions. 

**Figure 4:**
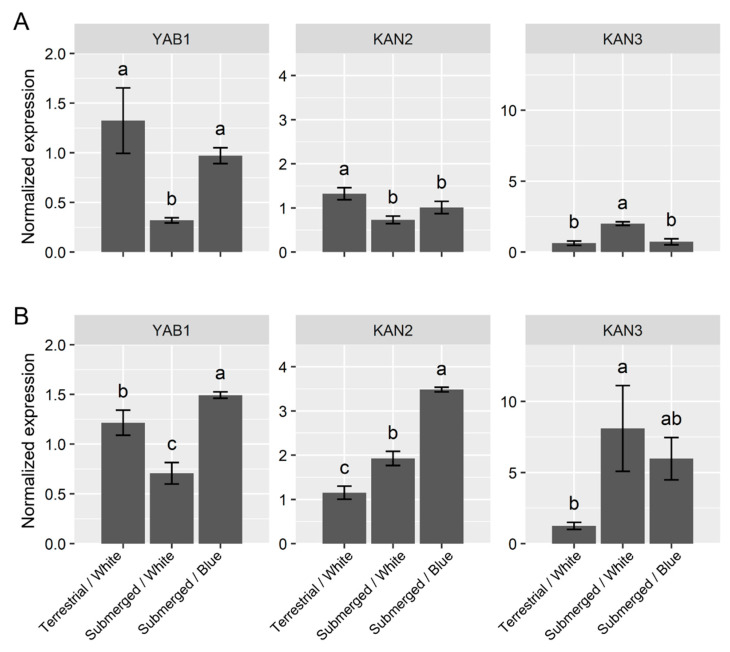
Expression profiles of the abaxialization genes after submergence. The plots indicate the relative expression normalized by *ubiquitin5* (mean ± SE, *n* = 3) after 1 day (**A**) and 7 days treatment (**B**). The different letters denote significant differences among the experimental conditions (Tukey–Kramer test; the adjusted *p*-value < 0.05).

The expression of the abaxialization-related genes *YABBY1* (*YAB1*) and *KANDAI2* (*KAN2*) was repressed by one day of submergence; this was similar to the adaxialization genes under the white-light conditions (Figure 4A). However, *KAN3* exhibited a different response to submergence. Its expression was upregulated under the submerged conditions. Although each abaxialization gene showed different response patterns to submergence, blue light canceled the effect of submergence. Under blue light, their expression levels were similar to those under terrestrial conditions. Seven days of submergence suppressed *YAB* and promoted *KAN3* similar to that under one day of submergence; in addition, blue light tended to suppress this effect (Figure 4B). *KAN2* showed increased expression after seven days of submergence and increased expression under blue light.

## 3. Discussion

An increase in leaf thickness in response to submergence in *R. aquatica* was observed in this study. As the ratio of intercellular area in leaf section area is low, an increase in cell size seems to be the most dominant factor in leaf thickness alternation. Gene expression analysis also supported this hypothesis. An increase in cell size under *AN3* suppressed conditions has been reported as a compensatory effect in *A. thaliana* [14,17,18]. Severe inhibition of the AN3/GRF complex led to small leaves with low cell numbers, but increased cell size. In *R. aquatica*, the expression of *AN3* and some *GRFs* was downregulated under submergence. Suppression of the AN3/GRF pathway may play an important role in cell-size expansion in response to submergence in *R. aquatica*. 

In addition, suppression of the effect of submergence under blue-light conditions at the cellular level was observed in this study. Under blue-light conditions, leaf cell morphology showed terrestrial-like characteristics; even under submergence (Figure 1). A similar phenomenon has been reported in the amphibious fern *Marsilea quadrifolia* [11]. Blue and purple lights induced the development of aerial leaves under the submerged conditions. At the molecular level, blue light inhibited the downregulation of cell proliferation-related genes after one day of submergence. This clearly correlates with the morphological observations. While the importance of regulation of the AN3/GRF pathway has been suggested, the upstream mechanism regulating AN3/GRF expression has not been fully identified; this is the case even in *A. thaliana*. Although the mechanism by which submergence represses *AN3* expression and is canceled under blue-light conditions has not been elucidated in *R. aquatica*, the following factors might mediate submergence and light quality.

*AN3* regulation via the photoreceptor phytochrome B (PhyB) was recently reported as a candidate [6]. PhyB is activated by red light and deactivated by far-red light; which represses the activity of *PHYTOCHROME-INTERACTING FACTOR*s (*PIF*s) encoding bHLH transcription factors [19]. *AN3* is important for reducing leaf size during the shade-avoidance syndrome. Under shaded conditions, the *AN3* and cell proliferation-related genes were downregulated; this lead to a decrease in leaf size. The accumulation of the PIF7 protein caused suppression of AN3 binding to the promoter region of the target genes, including *AN3*; resulting in the suppression of the downstream *AN3*/*GRF* pathway. In addition, *PIF7* is involved in cell-size compensation under the *AN3* defective conditions. Compensated cell size expansion was suppressed under active PIF conditions, such as in the *phyB* mutant or with far-red irradiation. Considering that activated PhyB returns to its inactive form spontaneously without far-red light by a phenomenon called dark reversion, the lack of a red spectrum under blue-light conditions might activate the PIFs. However, *AN3* expression was not suppressed under the submerged blue-light conditions. The regulation of *AN3* expression upon submergence in *R. aquatica* is controlled by other factors. However, PIF-mediated suppression of compensated cell-size expansion might be related to the submergence response of *R. aquatica*. Although the blue-light suppression of *AN3* and *GRF*s was only found within one day of submergence, PIF accumulation under blue-light conditions caused suppression of cell-size expansion by compensation upon submergence. Continuous activation of PIF under blue light keeps cell size small, even under submergence.

Another candidate factor is the phytohormone ethylene. Ethylene functions as a submergence signal transductor in amphibious plants [2]. Exogenous ethylene treatment causes submerged-type leaves under terrestrial conditions in various amphibious plant species. In *R. aquatica*, an increase in the amount of ethylene causes submerged-type leaves [12]. The molecular pathway of ethylene in submerged response has not yet been elucidated; however, the relationship between ethylene and plant morphology has been studied in the context of shade-avoidance syndrome. For example, both shade and low blue light induce stem elongation; however, this is not the case in ethylene-insensitive tobacco mutants [20]. This suggests that cell elongation signals are mediated by ethylene and blue light upstream. Although the pathway between ethylene and blue light remains unclear, a subsequent study supports that blue light signals via the photoreceptor *CRYPTOCHROME*s (*CRY*s) regulate ethylene signals. The overexpression of *CRY1* in *Brassica napus* inhibited ethylene signaling by downregulating the ethylene biosynthesis-related genes 1-aminocyclopropane-1-carboxylate synthase 5 and 8 [21]. Considering that ethylene induces submerged-type leaves in *R. aquatica*, the enhancement of ethylene signaling by submergence and repression by blue light may modulate leaf cell morphology. However, it remains unknown whether the ethylene pathway regulates the AN3/GRF pathway. 

Changes in the expression of genes related to adaxial-abaxial polarity were also observed in *R. aquatica*. However, a clear direction for adaxialization or abaxialization has not yet been identified. Both adaxial- and abaxial-related genes, except for *KAN3*, showed decreased expression as early responses upon submergence. This differs from the abaxialization of *Ranunculus trichophyllus* under submergence [16]. As some adaxial–abaxial polarity genes showed similar expression profiles to those of *AN3* and *GRFs*, polarity-related genes might be integrated in the submergence response pathway. On the other hand, regulatory systems work in a species-specific manner.

This study suggests that leaf form alternation occurs upon submergence; it may occur through the AN3/GRF pathway. Light quality was also integrated into heterophylly as a regulator of the submergence signal. They indicated a similarity between heterophylly and shade-avoidance syndrome; and the possibility that the two phenomena share the same downstream pathway. Although shade-avoidance syndrome is a highly conserved mechanism affecting leaf morphology in plants, heterophylly in response to submergence is independently achieved in various taxa. Heterophylly upon submergence might evolve based on the integration of submergence signals into the shade-avoidance syndrome. 

## 4. Materials and Methods

### 4.1. Plant Materials

*Rorippa aquatica* plants (accession N) [22] were placed in a growth chamber at 30 °C under continuous light at 30 µmol photons m^−2^ s^−1^ supplied by a fluorescent lamp. For each treatment, plants regenerated from the leaf tip using a previously described method [23] were used.

To observe the response to submergence, four weak old seedlings were transplanted to hydroponic sponges (KYOWA Co., Ltd., Osaka, Japan, Hyponica Leaf Vegetable Panel) fixed at the bottom of cubic glass tanks (W25 × D25 × H25 cm; Kotobuki, Nara, Japan, Crystal Cube 250) at 25 °C under continuous light conditions, as shown in Figure S1. The water level was maintained below the sponge’s surface under terrestrial conditions. Under the submerged conditions, 4.5 L of water was added to the glass tanks; resulting in 7.2 cm of water depth to fully submerge the seedlings planted on the sponges. The youngest premature leaf from each plant was used to observe the leaf cell morphology. For the blue-light treatment, the plants were grown at continuous blue light at 40 µmol photons m^−2^ s^−1^ supplied by a LED lamp.

### 4.2. Microscopic Morphological Observations

The young leaves that emerged under the experimental conditions for 7 days (5–8 mm leaf length) were fixed with FAA fixative (ethanol:acetic acid:formaldehyde:distilled water = 10:1:2:7). The fixed samples were dehydrated using an ethanol series and embedded in Technovit resin (Kulzer and Co., Wehrheim, Germany). The samples were sectioned into 4 µm slices with a Microm HM 325 microtome (Thermo Fisher Scientific, Waltham, MA, USA) and stained with 0.1% toluidine blue. The samples obtained from ten biological replicate leaves were photographed under a microscope; and the leaf thickness, area of mesophyll cells and density of intercellular regions were determined using the ImageJ software version 1.52 (http://rsb.info.nih.gov/ij/, Bethesda, MD, USA). Statistical analyses among the experimental conditions were performed with the Tukey–Kramer method using the multcomp package [24] in an R environment. The data were presented as a box plot using ggplot2 [25] in R.

### 4.3. RNA Extraction and RT-qPCR Analysis

Six young leaves were harvested from six different individuals and flash-frozen in liquid nitrogen; in addition, the total RNA was purified using the RNeasy Plant Mini Kit with DNase I digestion (Qiagen, Hilden, Germany). The quantity and integrity of the RNA were checked using a spectrophotometer and Agilent 2100 bioanalyzer (Agilent, Santa Clara, CA, USA). Reverse transcription was performed using ReverTra Ace (Toyobo, Osaka, Japan). Quantitative PCR was conducted using a SYBR Fast qPCR kit (Kapa Biosystems, Wilmington, MA, USA) and QuantStudio 3 system (Thermo Fisher Scientific). Data were obtained from three technical and three biological replicates. The expression level of *R. aquatica ubiqutin5* was used as an internal control. The primer sequences are listed in Table S1. Expression values normalized by *ubiqutin5* in each biological replicate were used for the statistical analyses. The data were analyzed with the Tukey–Kramer method using a multcomp package [24] and presented as a bar plot using ggplot2 [25] in R.

## Supplementary Materials

The following supporting information can be downloaded from https://www.mdpi.com/article/10.3390/ijms231810401/s1.
