# Supplementary material for: Leaf Cell Morphology Alternation in Response to Environmental Signals in Rorippa aquatica
Source: Int J Mol Sci. 2022 Sep 8;23(18):10401. doi: 10.3390/ijms231810401 (PMC9498993; doi:10.3390/ijms231810401)
Supplement: Supplementary file 1 [file ijms-23-10401-s001.zip › Figure S1.pdf]

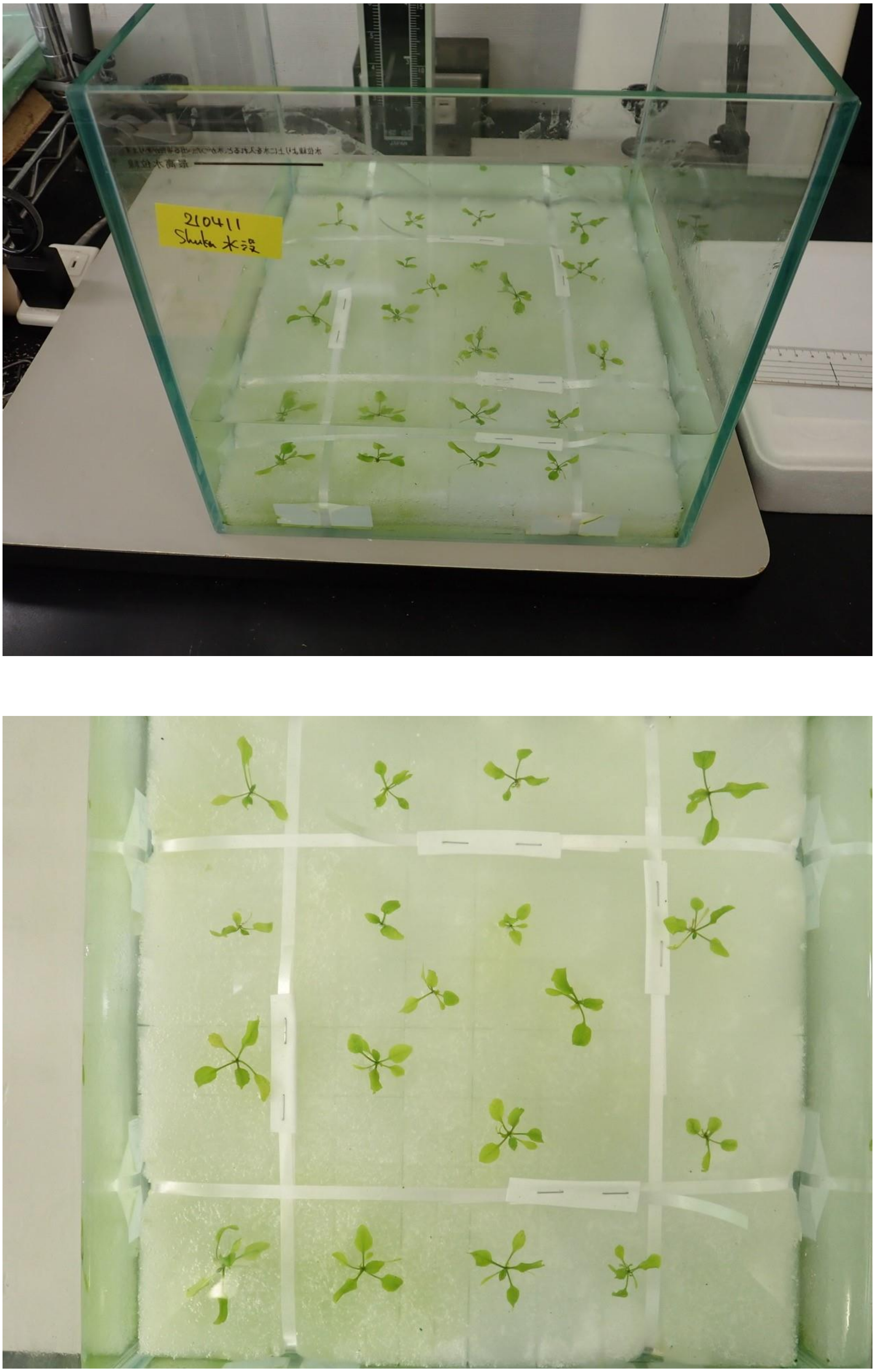

Figure S1. Experimental growth setting of *R. aquatica*. Side view (A) and top view (B) of submergence condition.
