# Supplementary material for: Leaf Cell Morphology Alternation in Response to Environmental Signals in Rorippa aquatica
Source: Int J Mol Sci. 2022 Sep 8;23(18):10401. doi: 10.3390/ijms231810401 (PMC9498993; doi:10.3390/ijms231810401)
Supplement: Supplementary file 1 [file ijms-23-10401-s001.zip › Table S1. Primer list for qPCR.pdf]

Supplementary Table S1. Primer list for qPCR.

name	Sequence (5'-3')
NK01_RaAN3-F	GGGAGAAGGAGCGTCACAC
NK02_RaAN3-R	CAACATTGAGGATCGAGCTGC
NK03_RaGRF2-F	CAGCCAATGTCCCTGTTCCT
NK04_RaGRF2-R	CCGAGATGGAAAGTTCCCCA
NK05_RaGRF4-F	CACCGTGGAAGAAACCGTTC
NK06_RaGRF4-R	TTGCTCTCTGACCAAGCTCC
NK07_RaAS2-F	ACCGTGAGAGACCACCACTA
NK08_RaAS2-R	GCAAGAATCCCACCGTCGTA
NK09_RaREV-F	TGAATCCGGTCGAAAGGCTC
NK10_RaREV-R	GTCGACACACAGATGCCTGA
NK11_RaPHB-F	CGATAGCAGAGGAGGCCCTA
NK12_RaPHB-R	AATCCGGACCAGGCTTCATC
NK13_RaYAB1-F	GCTCGTGCCAGACAATCAAC
NK14_RaYAB1-R	CGTTAGCCGTAGGAGCGTAG
NK15_RaKAN2-F	TTCCCAGCTAAGCGAAGCAT
NK16_RaKAN2-R	CTCTTTCATGGCCACCGAGT
NK17_RaKAN3-F	CACCGTAATCGTCGTCAAGC
NK18_RaKAN3-R	AAATGGGCATGAAGGGTCGT
